# Supplementary material for: Notch, RORC and IL-23 signals cooperate to promote multi-lineage human innate lymphoid cell differentiation
Source: Nat Commun. 2022 Jul 27;13:4344. doi: 10.1038/s41467-022-32089-3 (PMC9329340; doi:10.1038/s41467-022-32089-3)
Supplement: Supplementary file 1 — Supplementary information [file 41467_2022_32089_MOESM1_ESM.pdf]

Notch, RORC and IL-23 signals cooperate to promote multi-lineage human innate lymphoid cell differentiation

Carys A. Croft[1], Anna Thaller[1], Solenne Marie[1], Jean-Marc Doisne[1], Laura Surace[1], Rui Yang[2], Anne Puel[3,4,5], Jacinta Bustamante[3,4,5], Jean-Laurent Casanova[2,3,4,5,6] and James P. Di Santo[1,*]

Supplementary Information:

This file contains the following supplementary figures and tables:

- Supplementary Figure 1: Peripheral blood ILC identification and sorting strategy.
- Supplementary Figure 2: Impact of Notch and IL-23 signaling on human blood ILCP differentiation.
- Supplementary Figure 3: Analysis of blood ILCs and NK cells in *RORC*$^{-/-}$ patients.
- Supplementary Figure 4: Identification of ILC clones after human ILCP differentiation in vitro.
- Supplementary Figure 5: Impact of RORC inhibition on human ILCP differentiation.
- Supplementary Figure 6: Impact of IL-23 and Notch signaling on ILCP clonal differentiation.
- Supplementary Figure 7: Peripheral blood NK cells in IL12RB1$^{-/-}$ patients.
- Supplementary Figure 8: Impact of IL12RB1 deficiency on ILCP-derived clones.
- Supplementary Table 1
- Supplementary Table 2
- Supplementary Table 3
- Supplementary Table 4
- Supplementary Table 5
- Supplementary Table 6
- Supplementary Table 7
- Supplementary Table 8
- Supplementary Table 9

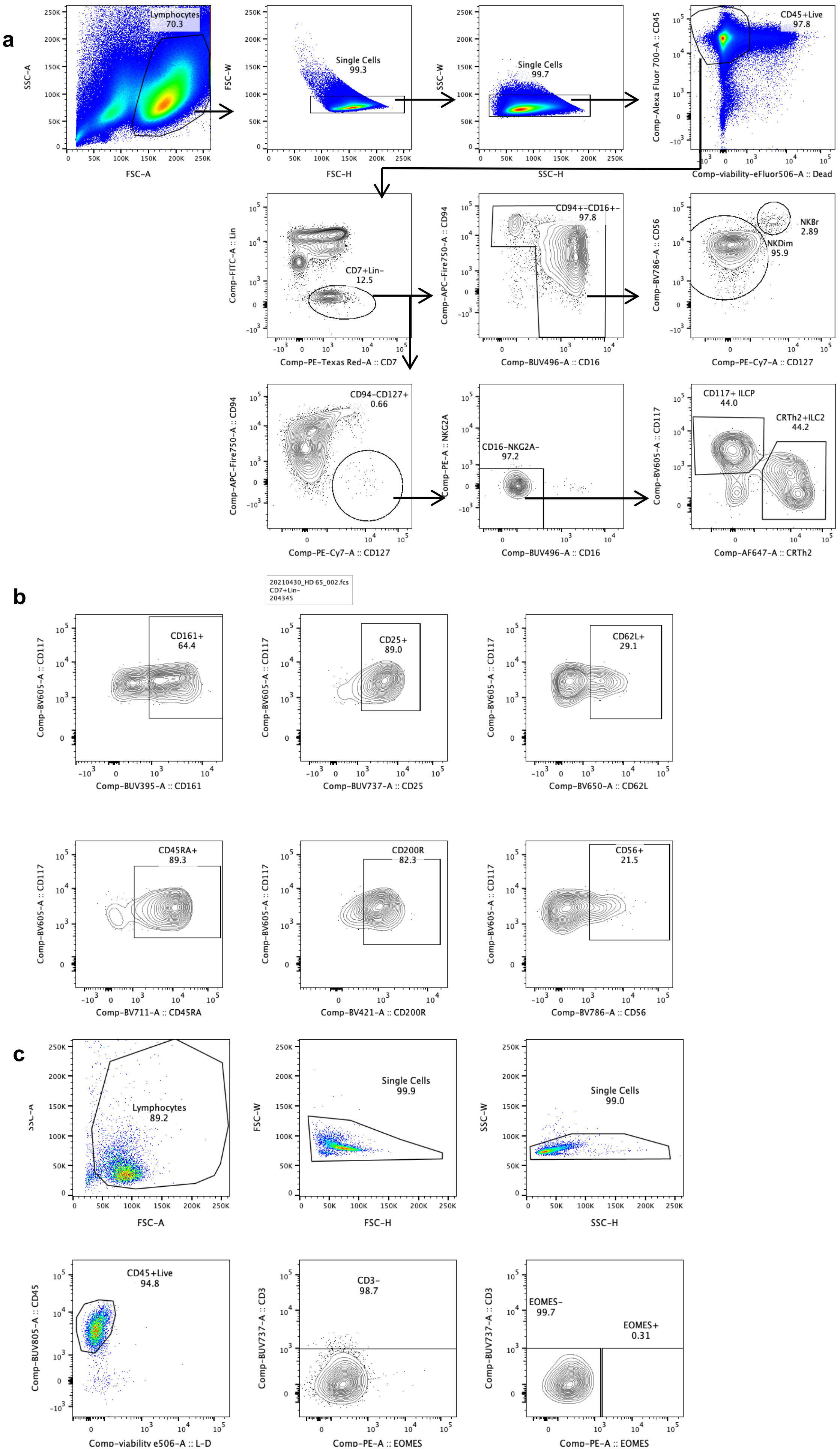

Supplementary Figure 1 - Peripheral blood ILC identification and sorting strategy.

(a) Identification of ILCs and NK Cells from CD45+Live lymphocytes excluding doublets. Lineage markers include CD3, CD4, CD5, CD14, CD19, TCRαβ and TCRγδ. Representative images of expression of (b) CD25, CD161, CD62L, CD56, CD45RA, CD200R on ILCP.

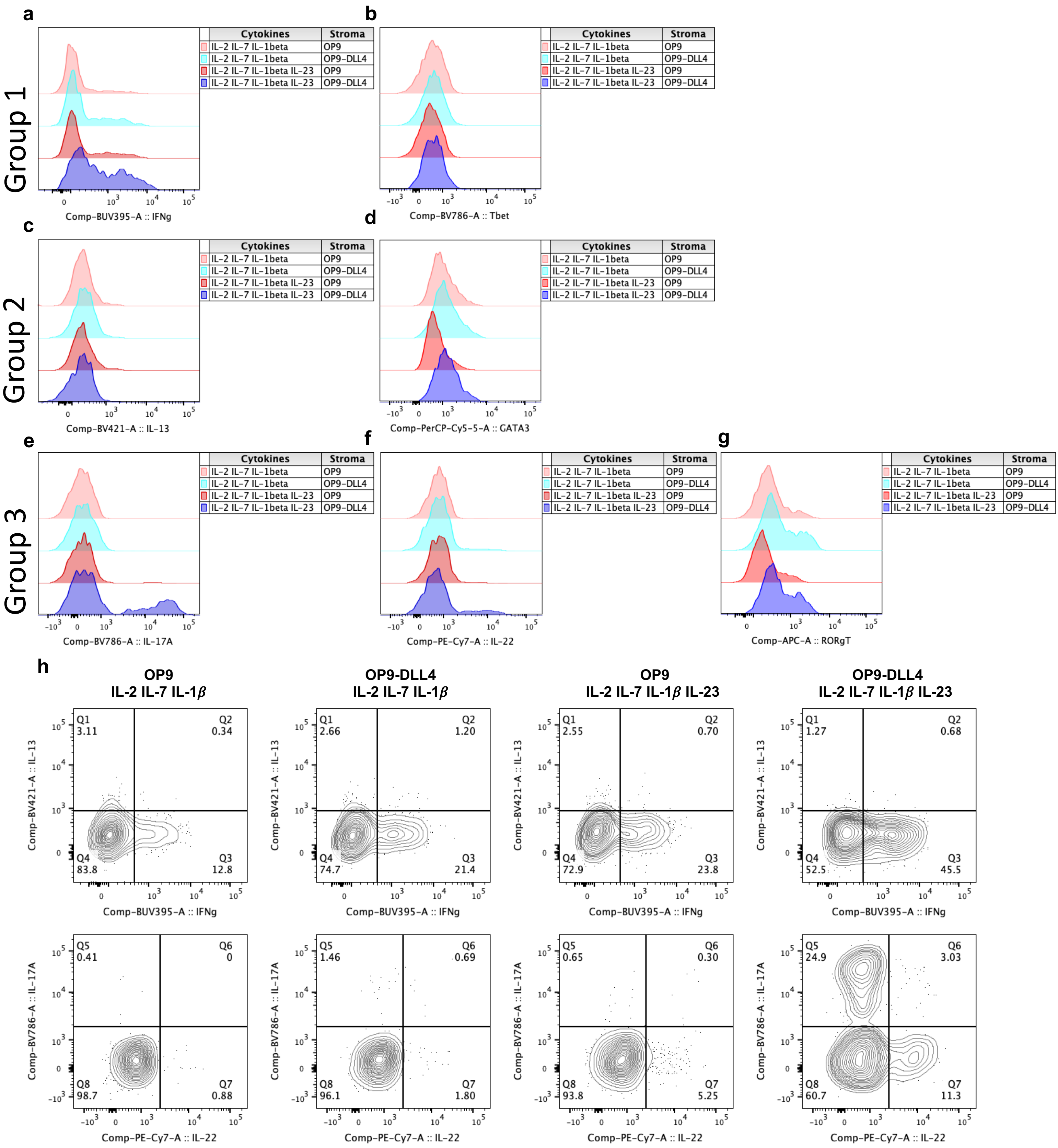

Supplementary Figure 2 - Impact of Notch and IL-23 signaling on human blood ILCP differentiation

Representative images of data from Fig. 1 comparing ILCP from the same donor grown on OP9 or OP9-DLL4 with or without IL-23 (10ng/ml). Histogram plots of (a) IFNγ, (b) T-bet, (c) IL-13, (d) GATA-3, (e) IL-17A, (f) IL-22, (g) RORγt expression. Representative scatterplots of cytokine expression from ILCP isolated from the same donor grown on (h) OP9 or OP9-DLL4 in the presence or absence of IL-23.

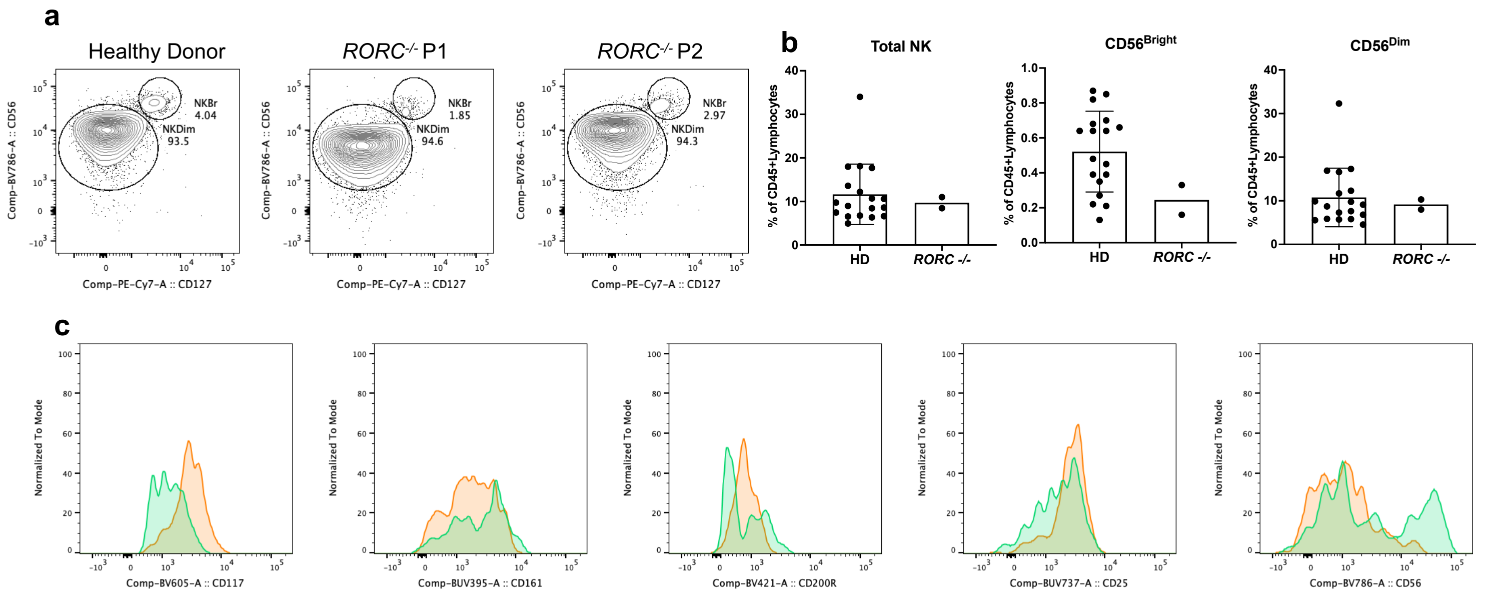

	Subset Name	Sample ID	Median : Comp-BV605-A	Median : Comp-BUV395-A	Median : Comp-BV421-A	Median : Comp-BUV737-A	Median : Comp-BV786-A
	CD117+ ILC	RORC	1232	2639	493	1788	1953
	CD117+ ILC	HD 002	2815	1725	760	2655	960

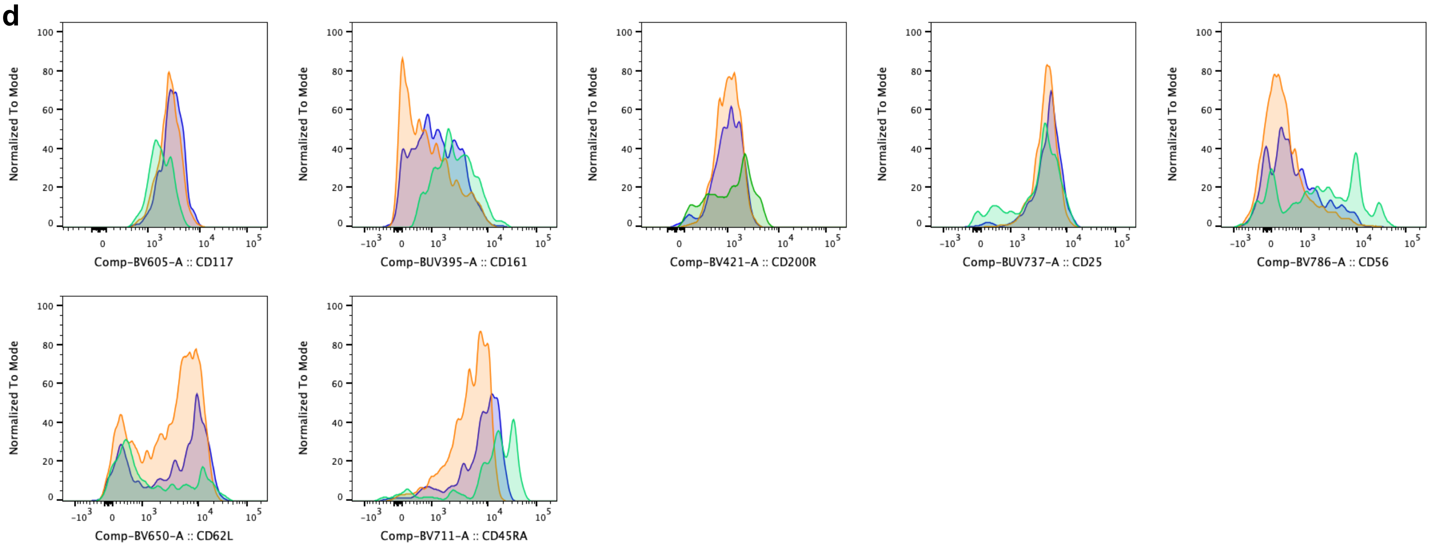

	Subset Name	SampleID	Median : Comp-BV605-A	Median : Comp-BUV395-A	Median : Comp-BV421-A	Median : Comp-BUV737-A	Median : Comp-BV786-A	Median : Comp-BV650-A	Median : Comp-BV711-A
	CD117+ ILC	RORC	1675	2283	1234	3626	2002	840	14618
	CD117+ ILC	HD 10	2461	703	1023	4082	336	3777	5039
	CD117+ ILC	HD 33	2793	1152	1057	4594	574	4903	8244

Supplementary Figure 3 - Analysis of blood ILCs and NK cells in RORC-/- patients.
(a) Representative images of CD56Bright and CD56Dim for one healthy donor and two RORC-/- patients gated on (CD16 and/or CD94)+Lin-CD7+CD45+live lymphocytes. (b) Relative frequencies of total NK cells, CD56Bright and CD56Dim in CD45+live lymphocytes. Data are represented as individual donors (n = 18) or patients (n = 2), mean with SD. (c-d) Representative images comparing healthy donors with RORC-/- patients for expression of the markers CD117, CRTh2, CD161, CD200R1, CD25 and CD56, as well as CD45RA and CD62L for P2, on ILCP from RORC-/- patients in the UMAPs found in Fig. 2. (c) Staining of P1 and (d) P2. Source Data are provided as a Source Data file.

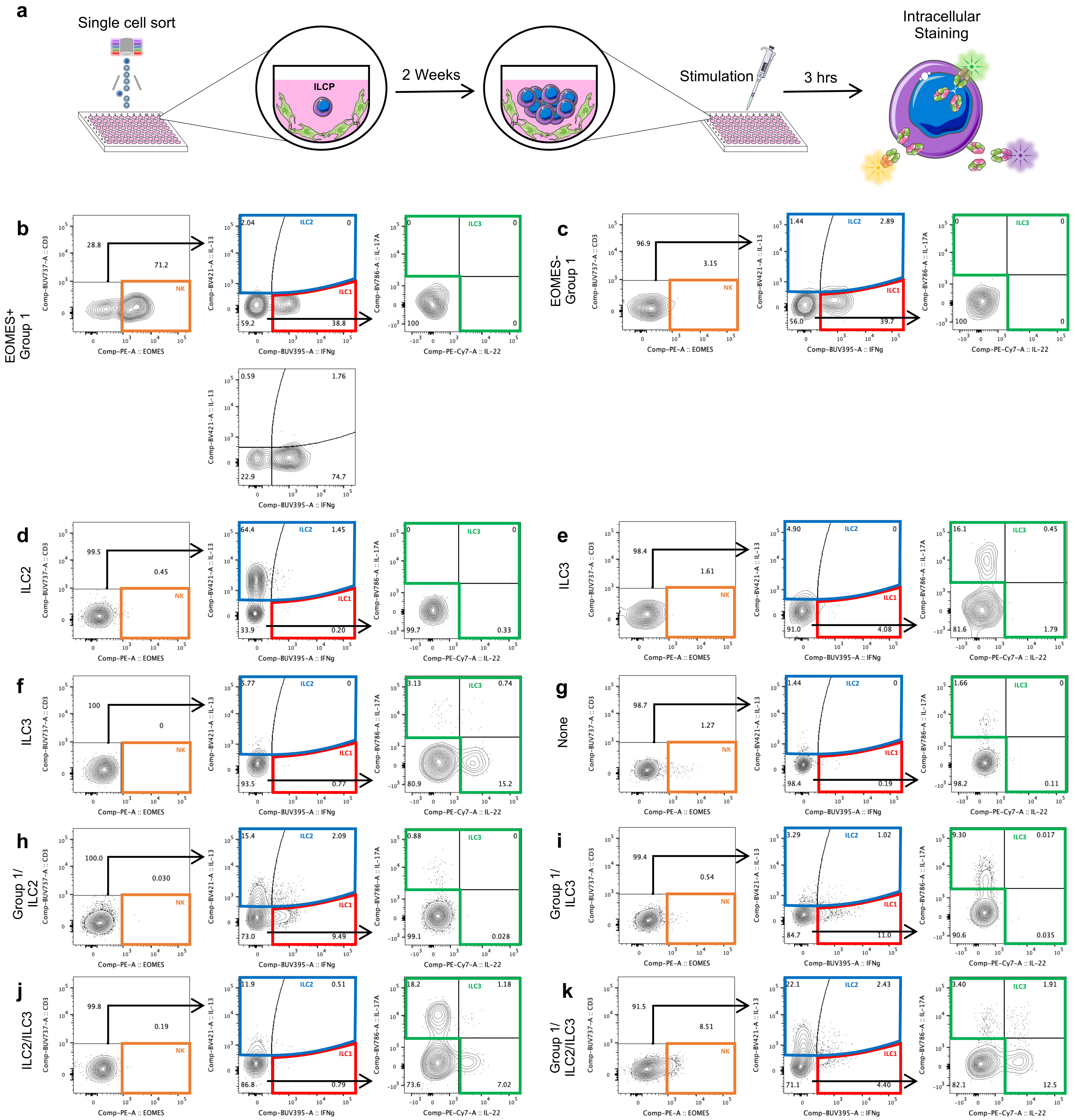

Supplementary Figure 4 - Identification of ILC clones after human ILCP differentiation in vitro.

(a) Cloning experiment workflow. ILCs are directly sorted into media containing stromal cells and cytokines, cultured for 2 weeks, selected, stimulated and then analyzed for cytokine expression. (b-k) Representative images of different types of clones. (b) EOMES+Group 1 clone, (c) EOMES-Group 2 clone, (d) ILC2, (e) IL-17A+ ILC3, (f) IL-22+ ILC3, (g) None, (h) Group 1/ILC2, (i) Group 1/ILC3, (j) ILC2/ILC3, (k) Group 1/ILC2/ILC3.

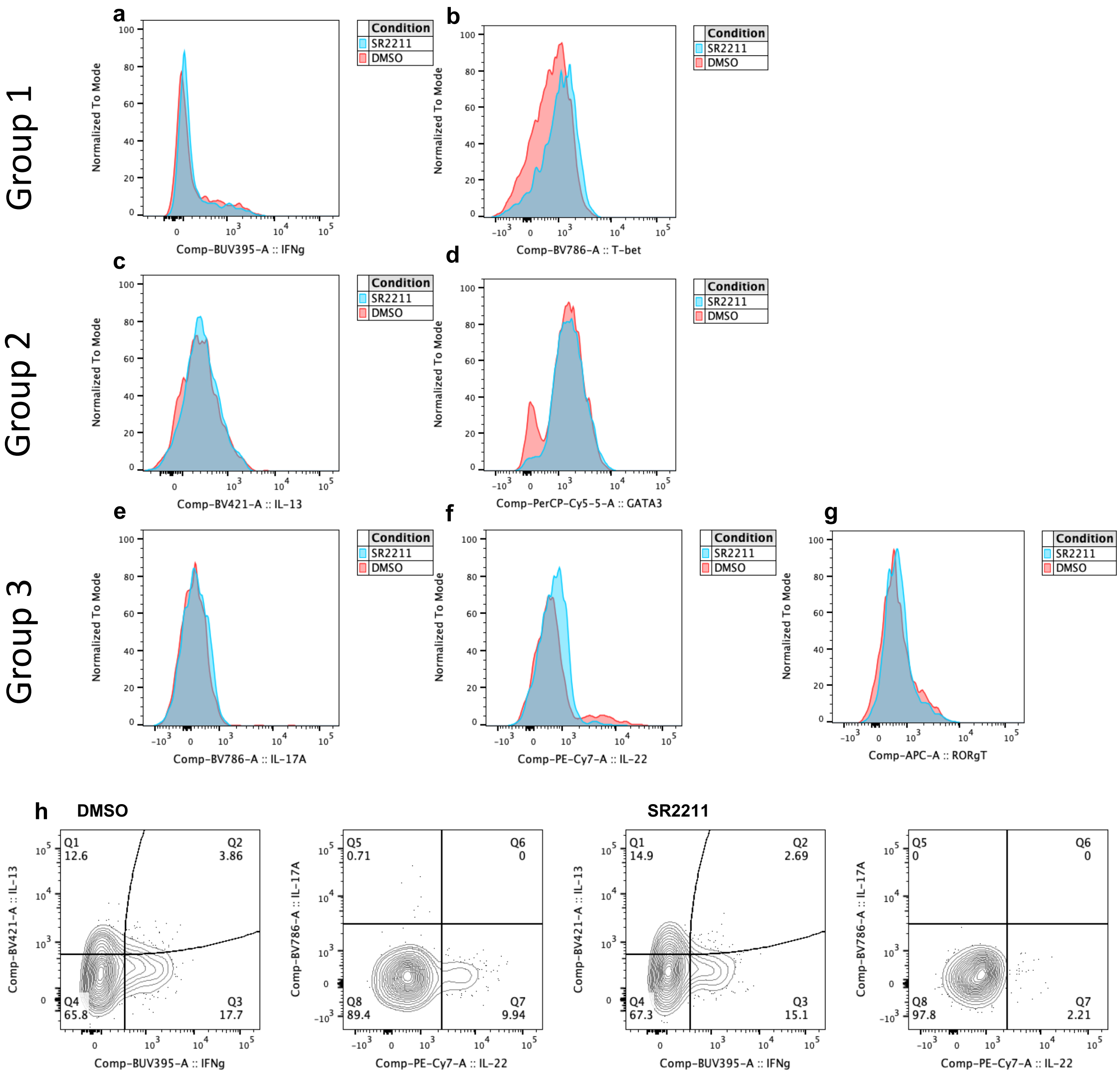

Supplementary Figure 5 - Impact of RORC inhibition on human ILCP differentiation.

Representative images of data from Fig. 3. ILCP from the same donor grown with either 0.01% DMSO or 10μM SR2211. Histogram plots of (a) IFNγ, (b) T-bet, (c) IL-13, (d) GATA-3, (e) IL-17A, (f) IL-22, (g) RORγt expression.

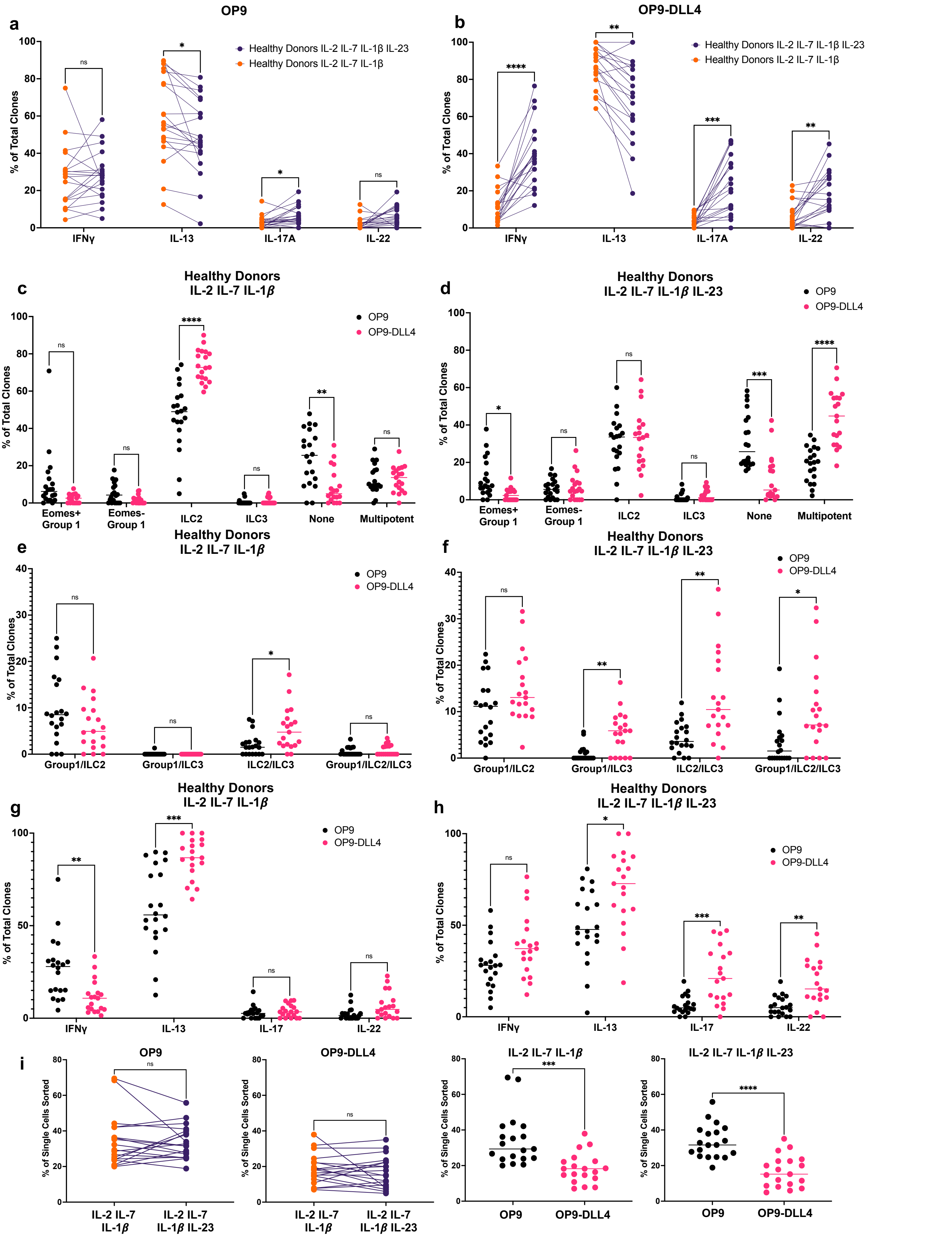

Supplementary Figure 6 - Impact of IL-23 and Notch signaling on ILCP clonal differentiation.
*(a-b) Frequencies of total clones expressing cytokines comparing the presence and absence of IL-23. ILCP grown on (a) OP9 (purple circles) or (b) OP9-DLL4 (orange circles). (c-h) Comparisons of healthy donors grown on OP9 (black circles) and OP9-DLL4 (pink circles). Frequencies of unipotent and total multipotent clones per individual healthy donor grown in (c) without or (d) with IL-23. Frequencies of types of multipotent clones grown in (e) without or (f) with IL-23. Frequencies of total cytokine producing clones grown in (g) without or (h) with IL-23. Comparisons performed using (a-h) Two-way ANOVA using Šidák's multiple comparisons test (a-b) with matching and (c-h) without, details in Supplementary Table 5. Cloning efficiency comparing (i) the presence or absence of IL-23 on both stroma compared by paired T-test or the presence of DLL4 on stroma compared by unpaired T-test (two-tailed), details in Supplementary Table 7. Data compiled from n = 20 biologically independent samples (OP9) or n = 19 biologically independent samples (OP9-DLL4) examined over a minimum of 7 independent experiments. Data are represented as individual donors either (a-b, i) paired or (c-h, j) unpaired. ns = not significant, * = p ≤ 0.05, ** = p ≤ 0.01, *** = p ≤ 0.001, **** = p < 0.001. Source Data are provided as a Source Data file.*

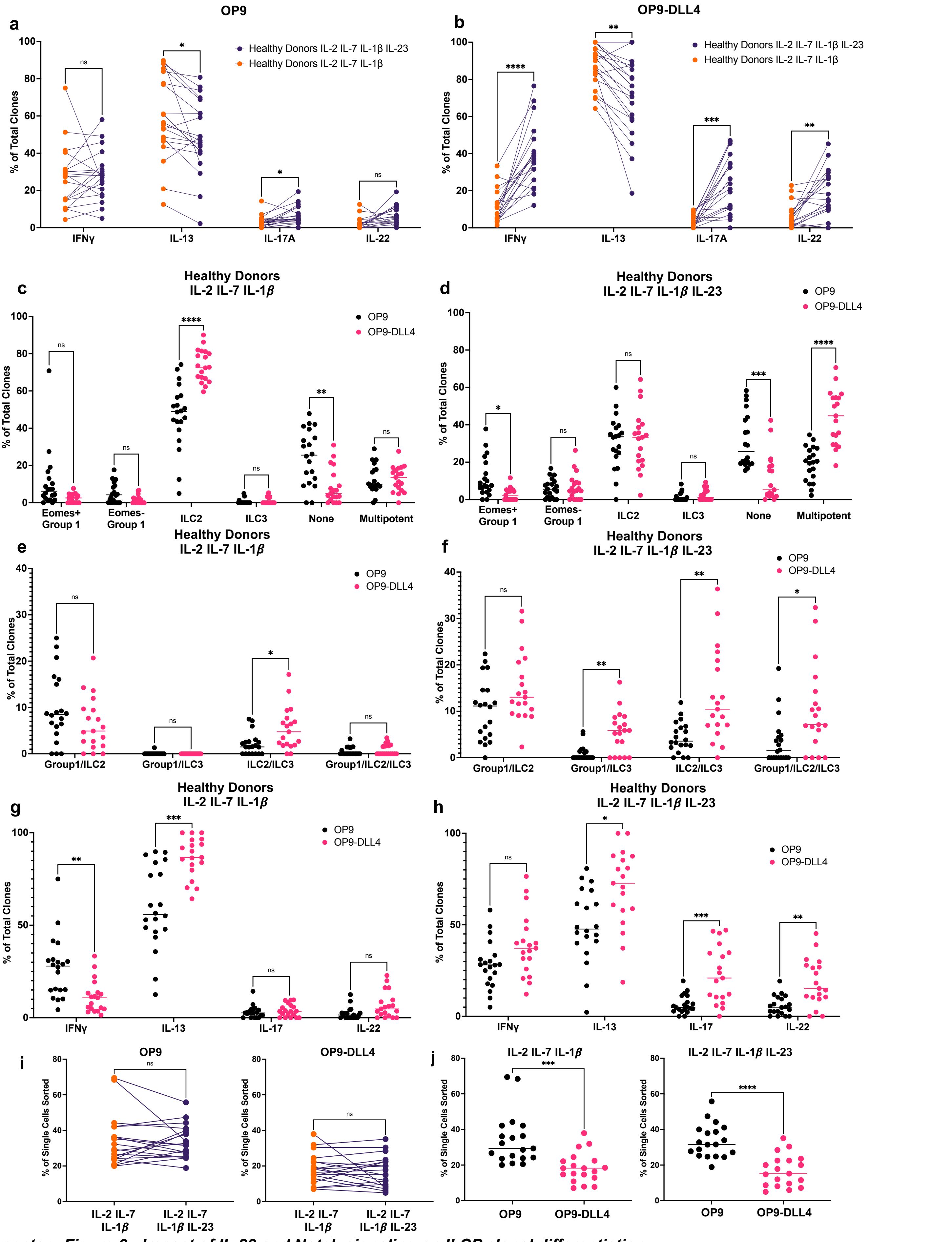

Supplementary Figure 6 - Impact of IL-23 and Notch signaling on ILCP clonal differentiation.

*(a-b) Frequencies of total clones expressing cytokines comparing the presence and absence of IL-23. ILCP grown on (a) OP9 (purple circles) or (b) OP9-DLL4 (orange circles). (c-h) Comparisons of healthy donors grown on OP9 (black circles) and OP9-DLL4 (pink circles). Frequencies of unipotent and total multipotent clones per individual healthy donor grown in (c) without or (d) with IL-23. Frequencies of types of multipotent clones grown in (e) without or (f) with IL-23. Frequencies of total cytokine producing clones grown in (g) without or (h) with IL-23. Comparisons performed using (a-h) Two-way ANOVA using Šidák's multiple comparisons test (a-b) with matching and (c-h) without, details in Supplementary Table 5. Cloning efficiency comparing (i) the presence or absence of IL-23 on both stroma compared by paired T-test or (j) the presence of DLL4 on stroma compared by unpaired T-test (two-tailed), details in Supplementary Table 7. Data compiled from n = 20 biologically independent samples (OP9) or n = 19 biologically independent samples (OP9-DLL4) examined over a minimum of 7 independent experiments. Data are represented as individual donors either (a-b, i) paired or (c-h, j) unpaired. ns = not significant, * = p ≤ 0.05, ** = p ≤ 0.01, *** = p ≤ 0.001, **** = p < 0.001. Source Data are provided as a Source Data file.*

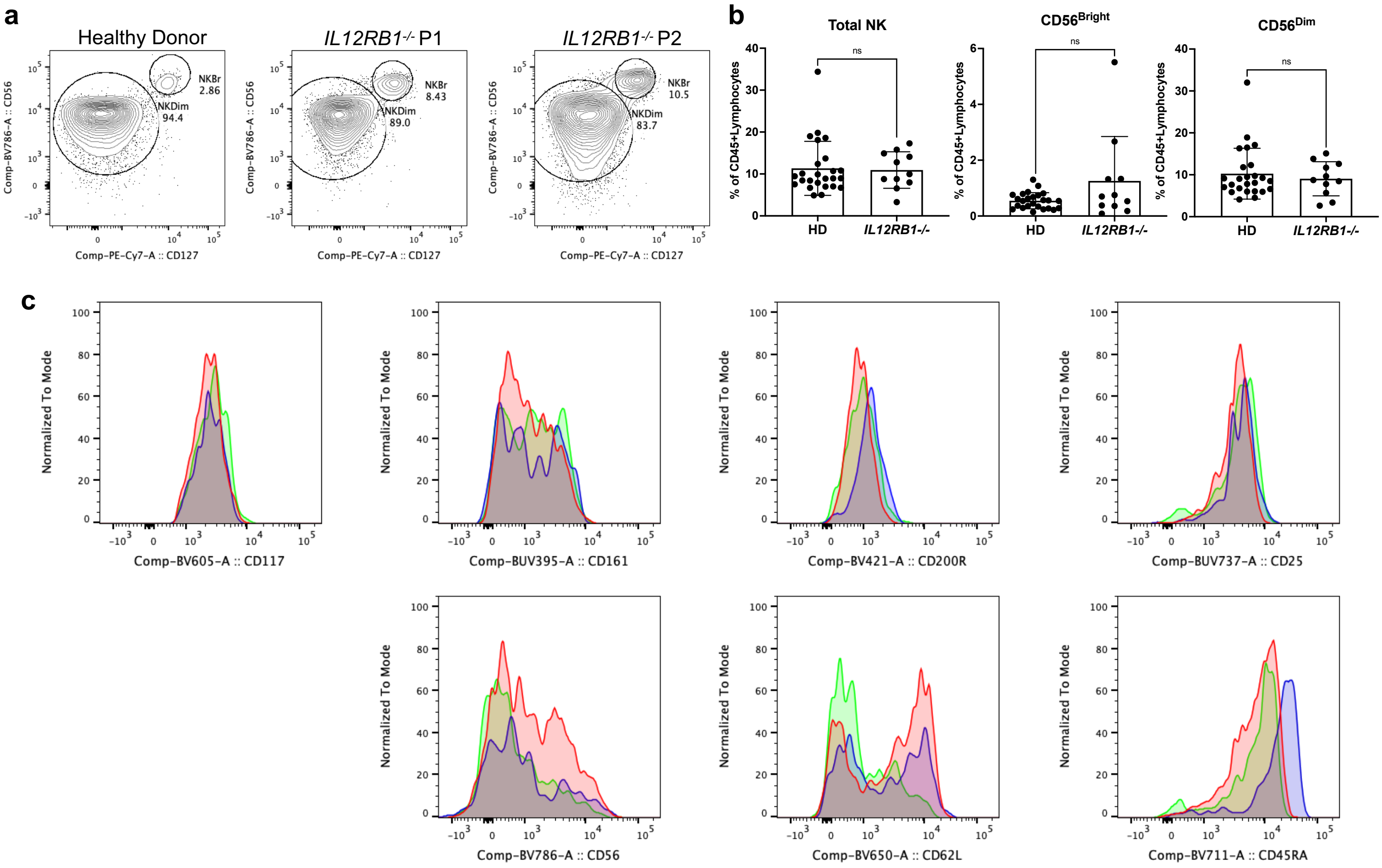

SampleID	Median : Comp-BV605-A	Median : Comp-BUV395-A	Median : Comp-BV421-A	Median : Comp-BUV737-A	Median : Comp-BV786-A	Median : Comp-BV650-A	Median : Comp-BV711-A
IL12RB1 1886	2206	1004	849	3214	998	3462	7765
IL12RB1 6657	2270	1022	1292	3983	668	2132	21310
HD 65	2624	1327	939	3863	443	641	8392

Supplementary Figure 7 - Peripheral blood NK cells in IL12RB1-/- patients.

*(a) Representative images of CD56Bright and CD56Dim for one healthy donor and two IL12RB1-/- patients gated on (CD16 and/or CD94)+Lin-CD7+CD45+live lymphocytes. (b) Relative frequencies of total NK cells, CD56Bright and CD56Dim in CD45+live lymphocytes. Comparisons performed using two-tailed Mann-Whitney test, details in Supplementary Table 8. ns = not significant, * = p ≤ 0.05, ** = p ≤ 0.01, *** = p ≤ 0.001, **** = p < 0.001. (c) Representative images comparing healthy donors with IL12RB1-/- patients for expression of the markers CD117, CRTh2, CD161, CD200R1, CD25, CD56, CD45RA and CD62L for ILCP from IL12RB1-/- patients in the UMAPs found in Fig. 6. Source Data are provided as a Source Data file.*

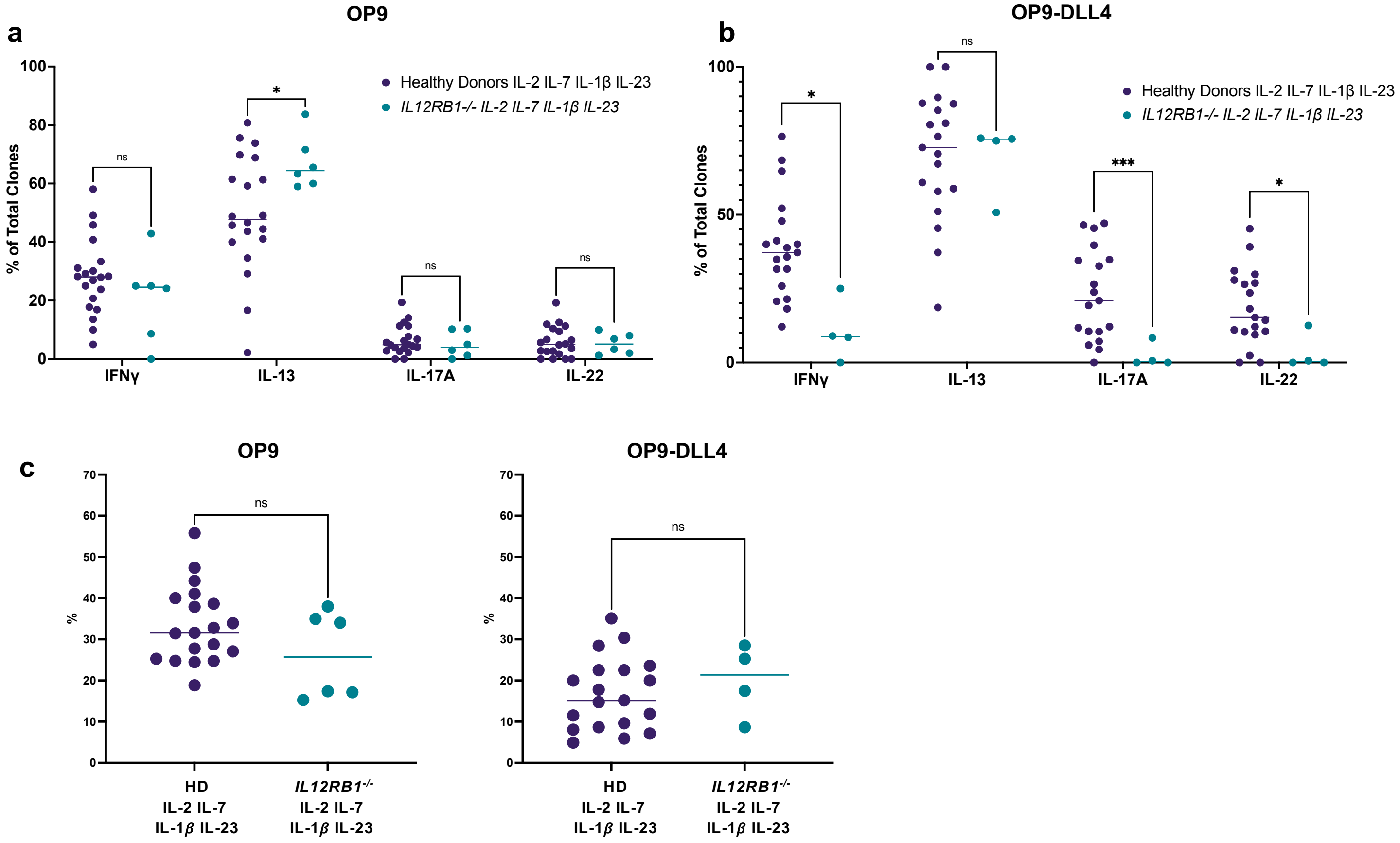

Supplementary Figure 8 - Impact of IL12RB1 deficiency on ILCP-derived clones.

*Single ILCP from healthy donors (purple circles) or IL12RB1-/- patients (green circles) were cultured on OP9 (HD, n = 20; IL12RB1-/-, n = 6) or OP9-DLL4 (HD, n = 19; IL12RB1-/-, n = 4) stroma. (a-b) Frequencies of total cytokine-producing clones on (a) OP9 (b) and OP9-DLL4. (a-b) Comparisons performed using Two-way ANOVA with no matching using Šidák's multiple comparisons test, details in Supplementary Table 5. ns = not significant, * = p ≤ 0.05, ** = p ≤ 0.01, *** = p ≤ 0.001, **** = p < 0.001. (c) Cloning efficiencies comparing ILCP from healthy donors or IL12RB1-/- patients, compared by unpaired T-test (details in Supplementary Table 7). Source Data are provided as a Source Data file.*

One Way Anova - Multiple comparisons

Figure	Column	Mean Diff	95,00% CI of diff	Below threshold?	Summary	Adjusted P Value	Mean 1	Mean 2	SE of diff,	n1	n2	q	DF
Figure 1a	A-B	-10.43	-16,64 to -4,222	Yes	***	0.0009	6.077	16.51	2.185	18	18	6.8	17
	A-C	-7.332	-14,50 to -0,1683	Yes	*	0.0439	6.077	13.41	2.52	18	18	4.1	17
	A-D	-16.64	-22,63 to -10,64	Yes	****	<0.0001	6.077	22.71	2.109	18	18	11	17
	B-C	3.101	-5,423 to 11,62	No	ns	0.7322	16.51	13.41	2.999	18	18	1.5	17
	B-D	-6.203	-14,37 to 1,968	No	ns	0.1751	16.51	22.71	2.874	18	18	3.1	17
	C-D	-9.304	-15,65 to -2,953	Yes	**	0.0033	13.41	22.71	2.234	18	18	5.9	17
Figure 1b	A-B	-24.31	-111,6 to 62,97	No	ns	0.8407	523.8	548.1	29.4	13	13	1.2	12
	A-C	-14.69	-73,41 to 44,03	No	ns	0.8779	523.8	538.5	19.78	13	13	1.1	12
	A-D	-65.46	-177,2 to 46,27	No	ns	0.3469	523.8	589.2	37.63	13	13	2.5	12
	B-C	9.615	-106,2 to 125,4	No	ns	0.9944	548.1	538.5	39	13	13	0.3	12
	B-D	-41.15	-191,8 to 109,5	No	ns	0.8482	548.1	589.2	50.76	13	13	1.1	12
	C-D	-50.77	-137,1 to 35,53	No	ns	0.3437	538.5	589.2	29.07	13	13	2.5	12
Figure 1d	A-B	2.198	0,1640 to 4,232	Yes	*	0.0317	6.219	4.021	0.7155	18	18	4.3	17
	A-C	-0.4056	-3,929 to 3,118	No	ns	0.9875	6.219	6.624	1.24	18	18	0.5	17
	A-D	1.25	-1,996 to 4,496	No	ns	0.6974	6.219	4.969	1.142	18	18	1.5	17
	B-C	-2.603	-5,927 to 0,7202	No	ns	0.1559	4.021	6.624	1.169	18	18	3.1	17
	B-D	-0.9477	-3,747 to 1,852	No	ns	0.7721	4.021	4.969	0.9848	18	18	1.4	17
	C-D	1.656	-0,3560 to 3,667	No	ns	0.128	6.624	4.969	0.7077	18	18	3.3	17
Figure 1e	A-B	105.7	-38,26 to 249,6	No	ns	0.184	884.4	778.7	48.49	13	13	3.1	12
	A-C	-146.2	-322,9 to 30,54	No	ns	0.1188	884.4	1031	59.52	13	13	3.5	12
	A-D	-124.2	-301,6 to 53,32	No	ns	0.2152	884.4	1009	59.78	13	13	2.9	12
	B-C	-251.8	-446,2 to -57,50	Yes	*	0.0108	778.7	1031	65.46	13	13	5.4	12
	B-D	-229.8	-423,0 to -36,67	Yes	*	0.0187	778.7	1009	65.07	13	13	5	12
	C-D	22	-51,83 to 95,83	No	ns	0.8128	1031	1009	24.87	13	13	1.3	12
Figure 1g	A-B	-0.291	-0,6074 to 0,0253	No	ns	0.0774	0.09122	0.3822	0.1113	18	18	3.7	17
	A-C	-0.1294	-0,3258 to 0,0670	No	ns	0.2759	0.09122	0.2206	0.0691	18	18	2.6	17
	A-D	-2.383	-6,667 to 1,902	No	ns	0.4149	0.09122	2.474	1.507	18	18	2.2	17
	B-C	0.1616	-0,09976 to 0,423	No	ns	0.3265	0.3822	0.2206	0.09195	18	18	2.5	17
	B-D	-2.092	-6,127 to 1,943	No	ns	0.4739	0.3822	2.474	1.42	18	18	2.1	17
	C-D	-2.253	-6,476 to 1,969	No	ns	0.4496	0.2206	2.474	1.485	18	18	2.1	17
Figure 1h	A-B	-0.3511	-1,071 to 0,3685	No	ns	0.5238	0.8811	1.232	0.2531	18	18	2	17
	A-C	-2.566	-4,447 to -0,6849	Yes	**	0.006	0.8811	3.447	0.6618	18	18	5.5	17
	A-D	-4.802	-7,070 to -2,534	Yes	****	<0.0001	0.8811	5.683	0.7979	18	18	8.5	17
	B-C	-2.215	-4,070 to -0,3595	Yes	*	0.0165	1.232	3.447	0.6528	18	18	4.8	17
	B-D	-4.451	-6,512 to -2,389	Yes	****	<0.0001	1.232	5.683	0.7251	18	18	8.7	17
	C-D	-2.236	-3,866 to -0,6054	Yes	**	0.0058	3.447	5.683	0.5735	18	18	5.5	17
Figure 1i	A-B	-2.558	-6,847 to 1,730	No	ns	0.3325	5.082	7.64	1.444	13	13	2.5	12
	A-C	-9.468	-14,34 to -4,591	Yes	***	0.0004	5.082	14.55	1.643	13	13	8.2	12
	A-D	-10.8	-14,59 to -7,002	Yes	****	<0.0001	5.082	15.88	1.278	13	13	12	12
	B-C	-6.909	-14,42 to 0,5985	No	ns	0.075	7.64	14.55	2.529	13	13	3.9	12
	B-D	-8.238	-14,10 to -2,373	Yes	**	0.0062	7.64	15.88	1.975	13	13	5.9	12
	C-D	-1.328	-5,220 to 2,564	No	ns	0.745	14.55	15.88	1.311	13	13	1.4	12
Supplementary Figure 6i	A-B	0	-7,740 to 7,740	No	ns	>0.9999	33.5	33.5	2.943	19	19	0	72
	A-C	16.76	9,023 to 24,50	Yes	****	<0.0001	33.5	16.73	2.943	19	19	8.1	72
	A-D	16.76	9,023 to 24,50	Yes	****	<0.0001	33.5	16.73	2.943	19	19	8.1	72
	B-C	16.76	9,023 to 24,50	Yes	****	<0.0001	33.5	16.73	2.943	19	19	8.1	72
	B-D	16.76	9,023 to 24,50	Yes	****	<0.0001	33.5	16.73	2.943	19	19	8.1	72
	C-D	0	-7,740 to 7,740	No	ns	>0.9999	16.73	16.73	2.943	19	19	0	72
Supplementary Figure 8c	A-B	8.043	-6,292 to 22,38	No	ns	0.4473	34.17	26.13	5.369	19	6	2.1	44
	A-C	15.69	5,757 to 25,62	Yes	***	0.0007	34.17	18.48	3.72	19	19	6	44
	A-D	14.19	-2,654 to 31,03	No	ns	0.126	34.17	19.98	6.307	19	4	3.2	44
	B-C	7.646	-6,690 to 21,98	No	ns	0.4915	26.13	18.48	5.369	6	19	2	44
	B-D	6.143	-13,62 to 25,90	No	ns	0.8399	26.13	19.98	7.401	6	4	1.2	44
	C-D	-1.503	-18,34 to 15,34	No	ns	0.9952	18.48	19.98	6.307	19	4	0.3	44

Supplementary Table 1 - *Details of multiple comparisons using one way Anova performed in Figure 1 and Supplementary Figures 6 and 8*

Wilcoxon Signed Rank Test (two-tailed)

Figure	Comparison	Theoretical Median	Actual Median	Significantly different (P < 0.05)?	P value summary	P value	Sum of signed ranks (W)	Sum of Positive ranks	Sum of negative ranks	Number of values	Discrepancy
Figure 1c (IL-2 IL-7 IL-1beta)	Comparison OP9 vs OP9-DLL4	1	0.9796	No	ns	0.8926	5	48	-43	13	-0.02037
Figure 1c (IL-2 IL-7 IL-1beta IL-23)	Comparison OP9 vs OP9-DLL4	1	1.113	No	ns	0.2334	32	55	-23	13	0.1135
Figure 1f (IL-2 IL-7 IL-1beta)	Comparison OP9 vs OP9-DLL4	1	1.14	Yes	*	0.0327	61	76	-15	13	0.14
Figure 1f (IL-2 IL-7 IL-1beta IL-23)	Comparison OP9 vs OP9-DLL4	1	1.426	Yes	**	0.0046	77	84	-7	13	0.4259
Figure 1j (IL-2 IL-7 IL-1beta)	Comparison OP9 vs OP9-DLL4	1	2.727	Yes	***	0.0002	91	91	0	13	1.727
Figure 1j (IL-2 IL-7 IL-1beta IL-23)	Comparison OP9 vs OP9-DLL4	1	2.241	Yes	***	0.0007	87	89	-2	13	1.241

***Supplementary Table 2 -** Details of Wilcoxon signed rank tests performed in Figure 1*

Supplementary Tables

Gene	Age at time of Sampling (years)	Country of Origin	Sex	Disease at moment of genetic diagnosis	Medication at the moment of sample testing	Mutation & Citation
**IL12RB1**	4	Morocco	M	MSMD (BCG-osis)	Multiple antimycobacterial antibiotics (no IFNg)	c.1791+2T>G/1791+2T>G66
**IL12RB1**	5	Greece	F	MSMD (BCG-osis)	None	c.1791+2T>G/deletion exon 15 to 1739
**IL12RB1**	37, 41	Morocco	M	BCG-osis, Salmonellosis at the moment of diagnosis (2003) since then free of infection	None	p.K305*/K305*67
**IL12RB1**	1.6	Morocco	F	BCG-itis	Multiple antimycobacterial antibiotics (no IFNg)	c.315_316del/315_316del
**IL12RB1**	3	Turkey	M	Disseminated infection by EM	Multiple antimycobacterial antibiotics (no IFNg)	p.T358*/T358*
**IL12RB1**	2	Turkey	F	BCG-itis, CMC	Antimycobacterial and antifungal drugs	p.R175W/R175W
**IL12RB1**	14	Iran	M	BCG-osis, invasive salmonellosis	Antibiotics (no IFNg)	homozygous large deletion (copy number variant) involving exons 1 to 739
**IL12RB1**	2	France	F	BCG-osis, CMC	None	p.G378R/Q32*
**IL12RB1**	13	France	F	Adenitis by M. avium	None	p.R486*/c.1791+2GT64
**IL12RB1**	22	France	F	BCG-osis	None	p.Q32*/Q32*66
**IL12RB1**	14	Turkey	M	BCG-itis, Salmonellosis	Antibiotics (no IFNg)	p.R211*/R211*68
**RORC**	8	Chile	F	BCG-osis, CMC	None	p.Q308*/Q308*27
**RORC**	11	India	F	Multifocal tuberculosis, CMC	Cotrimoxazole (antibiotics) daily prophylaxis	p.Y186*/Y186*

Supplementary Table 3 - *Details of patients used in this study*

Paired T-tests (two tailed)

Figure	Comparison	Mean of differences (B - A)	95% confidence interval	Significantly different (P < 0.05)?	P value summary	P value	SD of differences	SEM of differences	Number of pairs	df	t
Figure 3a	(A) DMSO vs (B) SR2211	-7.579	-10,34 to -4,818	Yes	***	0.0002	3.591	1.197	9	8	6.331
Figure 3c	(A) DMSO vs (B) SR2211	-1.312	-3,758 to 1,133	No	ns	0.2511	3.182	1.061	9	8	1.237
Figure 3e	(A) DMSO vs (B) SR2211	-0.6132	-0,9375 to -0,2890	Yes	**	0.0024	0.4218	0.1406	9	8	4.361
Figure 3f	(A) DMSO vs (B) SR2211	-3.384	-5,072 to -1,696	Yes	**	0.0017	2.196	0.732	9	8	4.624
Figure 3b	(A) DMSO vs (B) SR2211	103	-5,655 to 211,7	No	ns	0.061	171	49.37	12	11	2.086
Figure 3d	(A) DMSO vs (B) SR2211	-37.33	-161,6 to 86,89	No	ns	0.5219	195.5	56.44	12	11	0.662
Figure 3g	(A) DMSO vs (B) SR2211	-4.743	-8,652 to -0,8342	Yes	*	0.0218	6.152	1.776	12	11	2.671
Figure 4e	(A) DMSO vs (B) SR2211	-8.427	-10,85 to -6,002	Yes	****	<0.0001	4.55	1.137	16	15	7.408
Supplementary Figure 6i (OP9)	(A) - IL-23 vs (B) +IL-23	-0.6716	-6,275 to 4,932	No	ns	0.8041	11.63	2.667	19	18	0.252
Supplementary Figure 6i (OP9-DLL4)	(A) - IL-23 vs (B) +IL-23	-1.745	-6,059 to 2,568	No	ns	0.4065	8.949	2.053	19	18	0.85

***Supplementary Table 4 -** Details of paired t-tests performed in figures 3, 4 and supplementary figure 6*

Two Way Anova - Multiple comparisons

Figure	Group	Mean Diff	95.00% CI of diff	Below threshold?	Summary	Adjusted P Value	Mean 1	Mean 2	SE of diff	N1	N2	t	DF
Figure 4a	Eomes+ Group 1	0.08562	-3.198 to 3.369	No	ns	>0.9999	4.02	3.934	1.085	16	16	0.0789	15
	Eomes- Group 1	-1.614	-8.006 to 4.778	No	ns	0.9743	4.765	6.379	2.113	16	16	0.7639	15
	ILC2	-15.88	-28.58 to -3.171	Yes	*	0.0108	40.8	56.68	4.199	16	16	3.781	15
	ILC3	2.964	0.4878 to 5.440	Yes	*	0.015	3.994	1.031	0.8183	16	16	3.622	15
	None	-5.782	-12.69 to 1.127	No	ns	0.1303	14.54	20.32	2.283	16	16	2.532	15
	Multipotent	20.22	7.312 to 33.13	Yes	**	0.0016	31.88	11.66	4.267	16	16	4.739	15
Figure 4b	Group1/ILC2	0.0625	-4.432 to 4.557	No	ns	>0.9999	7.185	7.123	1.59	16	16	0.03931	15
	Group1/ILC3	5.343	2.204 to 8.481	Yes	***	0.0009	5.551	0.2081	1.11	16	16	4.812	15
	ILC2/ILC3	10.19	0.7713 to 19.61	Yes	*	0.0315	12.11	1.919	3.333	16	16	3.059	15
	Group1/ILC2/ILC3	4.626	-0.5328 to 9.784	No	ns	0.0884	7.034	2.408	1.825	16	16	2.535	15
Figure 4d	IFNγ	9.646	-0.4767 to 19.77	No	ns	0.065	26.92	17.27	3.581	16	16	2.694	15
	IL-13	-1.226	-15.75 to 13.29	No	ns	0.9988	67.94	69.17	5.136	16	16	0.2386	15
	IL-17	19.14	8.401 to 29.88	Yes	***	0.0006	22.14	3.002	3.799	16	16	5.039	15
	IL-22	10.5	3.812 to 17.19	Yes	**	0.0019	14.03	3.528	2.365	16	16	4.439	15
Figure 5a	Eomes+ Group 1	0.818	-9.023 to 10.66	No	ns	>0.9999	11.42	10.6	3.354	20	20	0.2439	19
	Eomes- Group 1	0.941	-1.375 to 3.257	No	ns	0.8189	6.037	5.096	0.7892	20	20	1.192	19
	ILC2	-16.05	-23.15 to -8.948	Yes	****	<0.0001	30.87	46.92	2.42	20	20	6.631	19
	ILC3	0.667	-1.180 to 2.514	No	ns	0.8848	1.162	0.4945	0.6293	20	20	1.06	19
	None	6.843	-3.242 to 16.93	No	ns	0.3148	31.35	24.51	3.437	20	20	1.991	19
	Multipotent	6.779	1.762 to 11.80	Yes	**	0.005	19.16	12.38	1.71	20	20	3.965	19
Figure 5b	Eomes+ Group 1	0.5053	-1.264 to 2.275	No	ns	0.9579	2.813	2.308	0.5992	19	19	0.8433	18
	Eomes- Group 1	5.163	0.9341 to 9.392	Yes	*	0.0121	6.78	1.617	1.432	19	19	3.605	18
	ILC2	-41.4	-51.52 to -31.29	Yes	****	<0.0001	32.24	73.64	3.426	19	19	12.09	18
	ILC3	1.572	-0.7856 to 3.929	No	ns	0.33	2.581	1.009	0.7983	19	19	1.969	18
	None	2.931	-3.920 to 9.782	No	ns	0.7793	11.54	8.61	2.32	19	19	1.263	18
	Multipotent	31.23	21.94 to 40.53	Yes	****	<0.0001	44.05	12.82	3.147	19	19	9.925	18
Figure 5c	Group1/ILC2	0.6695	-4.039 to 5.378	No	ns	0.9919	10.36	9.693	1.712	20	20	0.391	19
	Group1/ILC3	0.846	-0.2200 to 1.912	No	ns	0.1572	0.912	0.066	0.3877	20	20	2.182	19
	ILC2/ILC3	2.346	0.03955 to 4.651	Yes	*	0.0452	4.407	2.061	0.8386	20	20	2.797	19
	Group1/ILC2/ILC3	2.92	0.1399 to 5.692	Yes	*	0.0371	3.478	0.5615	1.01	20	20	2.888	19
Figure 5d	Group1/ILC2	8.59	3.110 to 14.06	Yes	**	0.0016	15.03	6.439	1.981	19	19	4.336	18
	Group1/ILC3	5.54	2.710 to 8.375	Yes	***	0.0002	5.543	0	1.024	19	19	5.411	18
	ILC2/ILC3	7.79	1.098 to 14.48	Yes	*	0.0189	13.27	5.478	2.42	19	19	3.219	18
	Group1/ILC2/ILC3	9.31	3.423 to 15.20	Yes	**	0.0015	10.21	0.8989	2.129	19	19	4.373	18
Supplementary Figure 6a	IFNγ	1.29	-8.267 to 10.85	No	ns	0.9934	28.09	26.8	3.476	20	20	0.3713	19
	IL-13	-9.67	-18.09 to -1.253	Yes	*	0.0205	49.64	59.31	3.062	20	20	3.159	19
	IL-17A	3.34	0.1608 to 6.510	Yes	*	0.04	6.42	3.08	1.16	20.00	20.00	2.89	19
	IL-22	3.59	-0.4655 to 7.637	No	ns	0.10	5.85	2.26	1.47	20.00	20.00	2.43	19
Supplementary Figure 6b	IFNγ	27.52	15.66 to 39.37	Yes	****	<0.0001	38.89	11.37	4.29	19.00	19.00	6.42	18
	IL-13	-16.62	-26.80 to -6.447	Yes	**	0.00	69.92	86.54	3.68	19.00	19.00	4.52	18
	IL-17A	18.90	9.010 to 28.78	Yes	***	0.00	22.81	3.91	3.58	19.00	19.00	5.29	18
	IL-22	11.78	3.321 to 20.25	Yes	**	0.00	18.60	6.82	3.06	19.00	19.00	3.85	18
Supplementary Figure 6c	Eomes+ Group 1	8.29	-2.165 to 18.75	No	ns	0.17	10.60	2.31	3.58	20.00	19.00	2.32	19.89
	Eomes- Group 1	3.48	-0.1683 to 7.127	No	ns	0.07	5.10	1.62	1.28	20.00	19.00	2.72	25.74
	ILC2	-26.72	-39.16 to -14.28	Yes	****	<0.0001	46.92	73.64	4.40	20.00	19.00	6.08	27.91
	ILC3	-0.515	-1.955 to 0.9255	No	ns	0.91	0.49	1.01	0.5149	20.00	19.00	1.00	33.16
	None	15.90	4.901 to 26.89	Yes	**	0.00	24.51	8.61	3.93	20.00	19.00	4.05	32.61
	Multipotent	-0.4353	-6.827 to 5.957	No	ns	>0.9999	12.38	12.82	2.3	20.00	19.00	0.19	36.92
Supplmentary Figure 6d	Eomes+ Group 1	8.61	1.647 to 15.56	Yes	*	0.01	11.42	2.81	2.42	20.00	19.00	3.56	22.93
	Eomes- Group 1	-0.74	-6.217 to 4.731	No	ns	1.00	6.04	6.78	1.96	20.00	19.00	0.38	32.63
	ILC2	-1.36	-14.76 to 12.03	No	ns	1.00	30.87	32.24	4.81	20.00	19.00	0.28	35.93
	ILC3	-1.42	-3.843 to 1.004	No	ns	0.51	1.16	2.58	0.87	20.00	19.00	1.64	33.08
	None	19.81	7.784 to 31.83	Yes	***	0.00	31.35	11.54	4.32	20.00	19.00	4.58	36.68
	Multipotent	-24.89	-35.88 to -13.90	Yes	****	<0.0001	19.16	44.05	3.90	20.00	19.00	6.38	30.27
Supplementary Figure 6e	Group1/ILC2	3.25	-2.310 to 8.818	No	ns	0.44	9.69	6.44	2.12	20.00	19.00	1.54	35.37
	Group1/ILC3	0.07	-0.1155 to 0.2475	No	ns	0.80	0.07	0.00	0.07	20.00	19.00	1.00	19
	ILC2/ILC3	-3.417	-6.549 to -0.2847	Yes	*	0.03	2.06	5.48	1.17	20.00	19.00	2.92	26.47
	Group1/ILC2/ILC3	-0.3374	-12.13 to 0.5379	No	ns	0.78	0.56	0.90	0.33	20.00	19.00	1.01	33.85
Supplementary Figure 6f	Group1/ILC2	-4.664	-10.57 to 1.245	No	ns	0.17	10.36	15.03	2.25	20.00	19.00	2.07	36.01
	Group1/ILC3	-4.631	-7.579 to -1.682	Yes	**	0.00	0.91	5.54	1.09	20.00	19.00	4.25	22.73
	ILC2/ILC3	-8.863	-15.32 to -23.51	Yes	**	0.01	4.41	13.27	2.40	20.00	19.00	3.70	21.72
	Group1/ILC2/ILC3	-6.731	-13.22 to -0.2385	Yes	*	0.04	3.48	10.21	2.44	20.00	19.00	2.76	27.44
Supplementary Figure 6g	IFNγ	15.42	-4.171 to 26.68	Yes	**	0.00	26.80	11.37	4.24	20.00	19.00	3.64	23.13
	IL-13	-27.23	-42.18 to -12.28	Yes	***	0.00	59.31	86.54	5.62	20.00	19.00	4.85	24.84
	IL-17A	-0.8296	-3.671 to 2.012	No	ns	0.91	3.08	3.91	1.09	20.00	19.00	0.76	36.64
	IL-22	-4.556	-9.353 to 0.2397	No	ns	0.07	2.26	6.82	1.79	20.00	19.00	2.55	25.15
Supplementary Figure 6f	IFNγ	-10.8	-23.70 to 2.099	No	ns	0.13	28.09	38.89	4.90	20.00	19.00	2.20	33.62
	IL-13	-20.28	-37.75 to -2.813	Yes	*	0.02	46.64	69.92	6.67	20.00	19.00	3.04	36.41
	IL-17A	-16.39	-26.47 to -6.313	Yes	***	0.00	6.42	22.81	3.11	20.00	19.00	4.42	21.52
	IL-22	-12.75	-21.28 to -4.228	Yes	**	0.00	5.85	18.60	3.16	20.00	19.00	4.03	23.53
Figure 7a	Eomes+ Group 1	10.01	2.974 to 17.04	Yes	**	0.00	11.42	1.41	2.44	20.00	6.00	4.10	22.74
	Eomes- Group 1	3.42	-1.162 to 8.009	No	ns	0.22	6.04	2.61	1.54	20.00	6.00	2.22	17.01
	ILC2	-21.07	-37.79 to -4.337	Yes	*	0.01	30.87	51.94	5.26	20.00	6.00	4.01	11.22
	ILC3	0.42	-1.905 to 2.745	No	ns	1.00	1.16	0.74	0.76	20.00	6.00	0.55	13.87
	None	3.14	-9.851 to 16.12	No	ns	0.98	31.35	28.21	4.38	20.00	6.00	0.72	17.54
	Multipotent	4.08	-14.22 to 22.37	No	ns	0.97	19.16	15.08	5.08	20.00	6.00	0.80	7.088
Figure 7b	Eomes+ Group 1	0.50	-15.01 to 16.01	No	ns	>0.9999	2.81	2.32	5.80	19.00	4.00	0.09	126
	Eomes- Group 1	4.01	-11.50 to 19.51	No	ns	0.98	6.78	2.77	5.80	19.00	4.00	0.69	126
	ILC2	-27.50	-43.01 to -12.00	Yes	****	<0.0001	32.24	59.74	5.80	19.00	4.00	4.74	126
	ILC3	1.54	-13.97 to 17.05	No	ns	>0.9999	2.58	1.04	5.80	19.00	4.00	0.27	126
	None	-13.03	-28.54 to 2.478	No	ns	0.15	11.54	24.57	5.80	19.00	4.00	2.25	126
	Multipotent	34.48	18.98 to 49.99	Yes	****	<0.0001	44.05	9.57	5.80	19.00	4.00	5.94	126
Figure 7c	Group1/ILC2	1.67	-10.64 to 13.99	No	ns	0.99	10.36	8.69	3.71	20.00	6.00	0.45	7.003
	Group1/ILC3	0.91	-0.1154 to 1.939	No	ns	0.09	0.91	0.00	0.37	20.00	6.00	2.44	19
	ILC2/ILC3	0.28	-2.463 to 3.016	No	ns	1.00	4.41	4.13	0.99	20.00	6.00	0.28	17.88
	Group1/ILC2/ILC3	1.22	-3.145 to 5.577	No	ns	0.91	3.48	2.26	1.57	20.00	6.00	0.78	16.91
Figure 7d	Group1/ILC2	8.89	-7.420 to 25.20	No	ns	0.30	15.03	6.14	4.03	19.00	4.00	2.21	4.407
	Group1/ILC3	5.54	2.710 to 8.375	Yes	***	0.00	5.54	0.00	1.02	19.00	4.00	5.41	18
	ILC2/ILC3	9.84	-2.793 to 22.47	No	ns	0.14	13.27	3.43	3.80	19.00	4.00	2.59	6.976
	Group1/ILC2/ILC3	10.21	4.252 to 16.16	Yes	***	0.00	10.21	0.00	2.15	19.00	4.00	4.74	18
Supplementary Figure 8a	IFNγ	7.15	-14.86 to 29.15	No	ns	0.79	28.09	20.94	6.76	20.00	6.00	1.06	7.46
	IL-13	-17.55	-33.64 to -1.457	Yes	*	0.03	49.64	67.19	5.85	20.00	6.00	3.00	19.03
	IL-17A	1.46	-5.118 to 8.029	No	ns	0.94	6.42	4.96	2.13	20.00	6.00	0.68	9.114
	IL-22	0.60	-4.820 to 6.018	No	ns	1.00	5.85	5.25	1.86	20.00	6.00	0.32	12.19
Supplementary Figure 8b	IFNγ	28.26	6.564 to 49.96	Yes	*	0.01	38.89	10.63	6.54	19.00	4.00	4.32	7.011
	IL-13	0.61	-25.10 to 26.33	No	ns	>0.9999	69.92	69.31	7.92	19.00	4.00	0.08	7.54
	IL-17A	20.57	9.360 to 31.79	Yes	***	0.00	22.81	2.24	4.08	19.00	4.00	5.04	19.24
	IL-22	15.33	2.339 to 28.31	Yes	*	0.02	18.60	3.28	4.26	19.00	4.00	3.60	9.638

Supplementary Table 5 - *Details of multiple comparisons with Two Way Anova performed in Figures 4, 5 and 7 and Supplementary Figures 6 and 8*

Chi-squared Test (two tailed)

Figure	Expected	Observed	Chi-square	DF	P value (two-tailed)	P value summary	Is discrepancy significant (P < 0.05)?
Figure 4c	DMSO	SR2211	141.5	8	<0.0001	****	Yes
Figure 5e	IL-2 IL-7 IL-1beta IL-23 (OP9)	IL-2 IL-7 IL-1beta (OP9)	187.8	8	<0.0001	****	Yes
Figure 5f	IL-2 IL-7 IL-1beta IL-23 (OP9-DLL4)	IL-2 IL-7 IL-1beta (OP9-DLL4)	616.2	8	<0.0001	****	Yes
Figure 7e	Healthy Donors (OP9)	IL12RB1-/- (OP9)	72.74	8	<0.0001	****	Yes
Figure 7f	Healthy Donors (OP9-DLL4)	IL12RB1-/- (OP9-DLL4)	218.8	8	<0.0001	****	Yes

Supplementary Table 6 - *Details of chi-squared tests performed in Figures 4, 5 and 7*

Unpaired T-test (two tailed)

Figure	Comparison	Mean of column A	Mean of column B	Significantly different (P < 0.05)?	P value summary	P value	Difference between means (B - A) ± SEM	95% confidence interval	Sample size, column A	Sample size, column B	df	t
Supplementary Figure 6j (-IL-23)	(A) OP9 vs (B) OP9-DLL4	34.17	18.48	Yes	***	0.0002	-15,69 ± 3,821	-23,44 to -7,940	19	19	36	4
Supplementary Figure 6j (+IL-23)	(A) OP9 vs (B) OP9-DLL4	33.5	16.73	Yes	****	<0.0001	-16,76 ± 2,943	-22,73 to -10,79	19	19	36	6
Supplementary Figure 8c (OP9)	(A) HD vs (B) IL12RB1-/-	33.5	26.13	No	ns	0.1152	-7,372 ± 4,503	-16,69 to 1,943	19	6	23	2
Supplementary Figure 8c (OP9-DLL4)	(A) HD vs (B) IL12RB1-/-	16.73	19.98	No	ns	0.5096	3,248 ± 4,842	-6,822 to 13,32	19	4	21	1

Supplementary Table 7 - *Details of unpaired t-tests performed in Supplementary Figures 6 and 8*

Mann Whitney test (two tailed)

Figure	Comparison	Difference: Actual	Difference: Hodges-Lehmann	Exact or approximate P value?	P value	Significantly different (P < 0.05)?	P value summary	Median of column A	Median of column B	N (column A)	N (column B)	Sum of ranks in column A,B	Mann-Whitney U
Figure 6b	HD vs IL12RB1-/-	0.097	0.07	Exact	0.0751	No	ns	0.093	0.19	25	11	410,5 , 255,5	85.5
Figure 6c	HD vs IL12RB1-/-	0.004	0	Exact	>0.9999	No	ns	0.044	0.048	25	11	462,5 , 203,5	137.5
Figure 6d	HD vs IL12RB1-/-	0.032	0.023	Exact	0.0156	Yes	*	0.024	0.056	25	11	393 , 273	68
Figure 6f	HD vs IL12RB1-/-	-14.2	-12.8	Exact	0.063	No	ns	44.2	30	25	11	517 , 149	83
Figure 6g	HD vs IL12RB1-/-	10.1	9.4	Exact	0.1235	No	ns	27.8	37.9	25	11	417 , 249	92
Figure 6e	HD vs IL12RB1-/-	-0.7332	-0.6324	Exact	0.0581	No	ns	1.516	0.783	25	11	518 , 148	82
Figure 6h (CD62L)	HD vs IL12RB1-/-	-3.65	7.2	Exact	0.7619	No	ns	64	60.35	6	4	31 , 24	10
Figure 6h (CD45RA)	HD vs IL12RB1-/-	4.25	3.9	Exact	0.0095	Yes	**	90.6	94.85	6	4	21 , 34	0
Figure 6h (CD200R1)	HD vs IL12RB1-/-	-2.6	-2.5	Exact	0.2799	No	ns	89	86.4	11	4	97 , 23	13
Figure 6h (CD161)	HD vs IL12RB1-/-	0.85	2.15	Exact	0.5714	No	ns	63.6	64.45	11	4	83 , 37	17
Figure 6h (CD25)	HD vs IL12RB1-/-	-0.1	1	Exact	0.9495	No	ns	90.3	90.2	11	4	87 , 33	21
Figure 6h (CD56)	HD vs IL12RB1-/-	-5.5	-5.95	Exact	0.7531	No	ns	40.8	35.3	11	4	91 , 29	19
Supplementary Figure 7b (Total NK)	HD vs IL12RB1-/-	1.01	0.4	Exact	0.8392	No	ns	8.96	9.97	25	11	456 , 210	131
Supplementary Figure 7b (CD56 Bright)	HD vs IL12RB1-/-	0.16	0.16	Exact	0.3119	No	ns	0.54	0.7	25	11	432,5 , 233,5	107.5
Supplementary Figure 7b (CD56 Dim)	HD vs IL12RB1-/-	0.22	-0.27	Exact	0.8658	No	ns	8.51	8.73	25	11	468 , 198	132

***Supplementary Table 8 -** Details of Mann Whitney tests performed in Figure 6 and Supplementary Figure 7*

Reagent	Fluorochrome/ Conjugate	Clone	Company	Cat. No.	Dilution
anti-CD45	PerCP-Vio700	5B1	Miltenyi Biotec	130-097-527	25
anti-CD45	AF700	HI30	BD Biosciences	560566	100
anti-CD45	BUV805	HI30	BD Biosciences	612891	200
anti-CD3	FITC	OKT3	Thermo Fisher Scientific	11-0037-42	50
anti-CD3	Biotin	OKT3	Thermo Fisher Scientific	13-0037-82	500
anti-CD3	BUV737	UCHT1	BD Biosciences	612750	100
anti-CD4	FITC	OKT4	BioLegend	317408	50
anti-CD5	FITC	UCHT2	Thermo Fisher Scientific	11-0059-42	50
anti-CD14	FITC	TÜK4	Miltenyi Biotec	130-080-701	50
anti-CD14	FITC	REA599	Miltenyi Biotec	130-110-518	100
anti-CD14	Biotin	61D3	Thermo Fisher Scientific	13-0149-82	500
anti-CD19	FITC	HIB19	BioLegend	302206	50
anti-CD19	FITC	REA675	Miltenyi Biotec	130-113-645	100
anti-CD19	Biotin	HIB19	Thermo Fisher Scientific	13-0199-82	500
anti-TCR$\alpha\beta$	FITC	IP26	Thermo Fisher Scientific	11-9986-42	50
anti-TCR$\gamma\delta$	FITC	B1	BD Biosciences	559878	50
anti-CRTH2	Alexa Fluor 647	BM16	BD Biosciences	558042	25
anti-CRTH2	PE	BM16	Miltenyi Biotec	130-091-238	12.5
anti-CD117	BV605	104D2	BioLegend	313218	20
anti-CD94	APC-Fire750	DX22	BioLegend	305518	25
anti-CD16	BV650	3G8	BioLegend	302042	100
anti-CD16	BUV496	3G8	BD Biosciences	564653	50
anti-NKG2A	PE	REA110	Miltenyi Biotec	130-113-566	500
anti-CD56	AF700	B159	BD Biosciences	557919	100
anti-CD56	BV785	5.1H11	BioLegend	362550	50
anti-CD127	PE-Cy7	eBioRDR5	Thermo Fisher Scientific	25-1278-42	100
anti-CD7	PE-CF594	M-T701	BD Biosciences	562841	50
anti-CD7	BV711	M-T701	BD Biosciences	564018	100
anti-CD7	BV650	M-T701	BD Biosciences	740565	100
anti-CD62L	BV650	DREG-56	BioLegend	304832	200
anti-CD25	BUV737	2A3	BD Biosciences	612806	100
anti-CD161	BUV395	HP-3G10	BD Biosciences	745737	12.5
anti-CD200R	BV421	OX-108	BD Biosciences	566344	50
anti-CD45RA	BV711	HI100	BioLegend	304138	200
anti-IFNγ	BUV395	B27	BD Biosciences	624163	200
anti-IL-13	BV421	JES10-5A2	BD Biosciences	624124	100
anti-IL-17A	BV785	BL168	BioLegend	512338	50
anti-IL-22	PE-Cy7	22URTI	Thermo Fisher Scientific	25-7229-42	25
anti-EOMES	PE-Cy7	WD1928	Thermo Fisher Scientific	25-4877-42/41	25
anti-EOMES	PE	WD1928	Thermo Fisher Scientific	12-4877-42	50
anti-RORγT	Alexa Fluor 647	Q21-559	BD Biosciences	563620	50
anti-GATA-3	PerCP-eFluor 71	TWAJ	Thermo Fisher Scientific	46-9966-42	50
anti-T-bet	BV786	O4-46	BD Biosciences	564141	25
anti-CD123	Biotin	6H6	Thermo Fisher Scientific	13-1239-82	500
anti-CD235a	Biotin	HIR2	Thermo Fisher Scientific	13-9987-82	500
Fixable Viability Dye	eFluor 506		Thermo Fisher Scientific	65-0866-14	200
Foxp3 / Transcription Factor Staining Buffer Set			Thermo Fisher Scientific	00-5523-00	
Brilliant Stain Buffer			BD Biosciences	563794	
Recombinant human IL-2			Miltenyi Biotec	130-097-743	
Recombinant human IL-7			Miltenyi Biotec	130-095-363	
Recombinant human IL-1β			Peprotech	200-01B	
Recombinant human IL-23			Peprotech	200-23	
SR2211			Tocris (Biotechne)	4869	
Golgi Plug			BD Biosciences	51-2301KZ	
Golgi Stop			BD Biosciences	51-2092KZ	
Ionomycin calcium			Sigma-Aldrich	I0634	
PMA			Sigma-Aldrich	P1585	

Supplementary Table 9 - *List of Reagents for flow cytometry and cell culture*
