# Supplementary material for: Notch, RORC and IL-23 signals cooperate to promote multi-lineage human innate lymphoid cell differentiation
Source: Nat Commun. 2022 Jul 27;13:4344. doi: 10.1038/s41467-022-32089-3 (PMC9329340; doi:10.1038/s41467-022-32089-3)
Supplement: Supplementary file 3 — Reporting Summary [file 41467_2022_32089_MOESM3_ESM.pdf]

nature portfolio

Corresponding author(s): James Di Santo

Last updated by author(s): Jun 9, 2022

Reporting Summary

Nature Portfolio wishes to improve the reproducibility of the work that we publish. This form provides structure for consistency and transparency in reporting. For further information on Nature Portfolio policies, see our Editorial Policies and the Editorial Policy Checklist.

Statistics

For all statistical analyses, confirm that the following items are present in the figure legend, table legend, main text, or Methods section.

n/a	Confirmed	
☐	☒	The exact sample size (*n*) for each experimental group/condition, given as a discrete number and unit of measurement
☐	☒	A statement on whether measurements were taken from distinct samples or whether the same sample was measured repeatedly
☐	☒	The statistical test(s) used AND whether they are one- or two-sided<br>*Only common tests should be described solely by name; describe more complex techniques in the Methods section.*
☒	☐	A description of all covariates tested
☐	☒	A description of any assumptions or corrections, such as tests of normality and adjustment for multiple comparisons
☐	☒	A full description of the statistical parameters including central tendency (e.g. means) or other basic estimates (e.g. regression coefficient) AND variation (e.g. standard deviation) or associated estimates of uncertainty (e.g. confidence intervals)
☐	☒	For null hypothesis testing, the test statistic (e.g. *F*, *t*, *r*) with confidence intervals, effect sizes, degrees of freedom and *P* value noted<br>*Give P values as exact values whenever suitable.*
☒	☐	For Bayesian analysis, information on the choice of priors and Markov chain Monte Carlo settings
☒	☐	For hierarchical and complex designs, identification of the appropriate level for tests and full reporting of outcomes
☒	☐	Estimates of effect sizes (e.g. Cohen's *d*, Pearson's *r*), indicating how they were calculated

Our web collection on statistics for biologists contains articles on many of the points above.

Software and code

Policy information about availability of computer code

Data collection	BD FACS DIVA v6 and v8
Data analysis	FlowJo v10.8.0, FlowJo Umap Plugin v3.1, Prism 9.2.0

For manuscripts utilizing custom algorithms or software that are central to the research but not yet described in published literature, software must be made available to editors and reviewers. We strongly encourage code deposition in a community repository (e.g. GitHub). See the Nature Portfolio guidelines for submitting code & software for further information.

Data

Policy information about availability of data

All manuscripts must include a data availability statement. This statement should provide the following information, where applicable:

- Accession codes, unique identifiers, or web links for publicly available datasets
- A description of any restrictions on data availability
- For clinical datasets or third party data, please ensure that the statement adheres to our policy

All data generated or analyzed during this study are available as Supplementary Data. Source data are provided with this paper.

Field-specific reporting

Please select the one below that is the best fit for your research. If you are not sure, read the appropriate sections before making your selection.

☒ Life sciences ☐ Behavioural & social sciences ☐ Ecological, evolutionary & environmental sciences

For a reference copy of the document with all sections, see nature.com/documents/nr-reporting-summary-flat.pdf

Life sciences study design

All studies must disclose on these points even when the disclosure is negative.

Sample size	No specific sample size calculation was performed. Sample sizes were determined in part based upon the availability of rare samples from patients with deleterious mutations and samples of healthy donors. Regarding healthy donors pairing was used to increase the power of the analysis where possible. Sample sizes are within or above norms of similar experiments done in the field. For experiments where statistics were performed, a minimum number of donors were used to achieve statistical significance (n>3), and a minimum of 3 independent experiments were performed. Stringent statistical testing including corrections for multiple comparisons were performed at each stage.
Data exclusions	Data was not excluded from the analysis
Replication	Experiments have been replicated independently a minimum of 3 times, the experimental procedure was performed and analyzed interdependently by first, second and third authors. Pairing was employed to account for genetic variation and differences in environmental exposure between healthy donors. Multiple healthy donors tested from frozen samples were single cell cloned at separate times across independent experiments to confirm reproducibility of results.
Randomization	Donors were acquired with no specific criteria from voluntary blood donations. No pre-selection was done for conditions tested or experiments done. Randomization is also not necessary for the majority of experiments as cultures from healthy donors were concurrently grown and analyzed in different conditions to account for normal genetic variation and reduce the effect of other covariates, such as environmental exposures. Concurrent analysis of different tested conditions was used to reduce technical variation.
Blinding	As stated above, donors were acquired with no specific criteria from voluntary blood donations. Blinding regarding the patients was not possible due to methodological constraints and the necessity of identifying the samples. Blinding of ICS analysis of clones and bulks was not possible due to the requirement for identifying the culture conditions and origin of each sample. Analysis was done simultaneously on clones from different conditions and the same gates and strategy were used to

Reporting for specific materials, systems and methods

We require information from authors about some types of materials, experimental systems and methods used in many studies. Here, indicate whether each material, system or method listed is relevant to your study. If you are not sure if a list item applies to your research, read the appropriate section before selecting a response.

Materials & experimental systems

n/a	Involved in the study	
☐	☒	Antibodies
☐	☒	Eukaryotic cell lines
☒	☐	Palaeontology and archaeology
☒	☐	Animals and other organisms
☐	☐	Human research participants
☐	☒	Clinical data
☐	☐	Dual use research of concern

Methods

n/a	Involved in the study	
☒	☐	ChIP-seq
☐	☒	Flow cytometry
☒	☐	MRI-based neuroimaging

Antibodies

Antibodies used	All antibodies used in the study are detailed in supplementary table 2, as well as dilutions.<br>Antibodies used for ILCP enrichment include:<br>anti-CD3-biotin (OKT3; 13-0037-82), 1:500,<br>anti-CD14-biotin (61D3; 13-0149-82), 1:500,<br>anti-CD19-biotin (HIB19; 13-0199-82), 1:500,<br>anti-CD123-biotin (6H6; 13-1239-82), 1:500,<br>anti-CD235a-biotin (HIR2; 13-9987-82) 1:500,<br>were purchased from Thermo Fisher Scientific.<br>Antibodies used for ILCP sorting, isolation, phenotyping and functional analysis include, purchased from Miltenyi Biotec:<br>anti-CD45-PerCP-Vio700 (5B1; 130-097-527), 1:25,<br>anti-CD14-FITC (TÜK4; 130-080-701, REA599; 130-110-518), 1:50,

anti-CD19-FITC (REA675; 130-113-645), 1:50,
anti-NKG2A-PE (REA110; 130-113-566), 1:500,
anti-CRTH2-PE (BM16; 130-091-238). 1:12.5,
Purchased from Biolegend:
anti-CD4-FITC (OKT4; 317408), 1:50,
anti-CD19-FITC (HIB19; 302206), 1:50,
anti-CD117-BV605 (104D2; 313218), 1:20,
anti-CD94-APC-Fire750 (DX22; 305518), 1:25,
anti-CD16-BV650 (3G8; 302042), 1:100,
anti-CD56-BV785 (5.1H11; 362550), 1:50,
anti-CD62L-BV650 (DREG-56; 304832), 1:200,
anti-CD45RA-BV711 (HI100; 304138), 1:200,
anti-IL-17A-BV785 (BL168; 512338) 1:50.
Purchased from BD Biosciences:
anti-CD45-AF700 (HI30; 560566), 1:100,
anti-CD45-BUV805 (HI30; 612891), 1:200,
anti-CD3-BUV737 (UCHT1; 612750), 1:100,
anti-TCRgd-FITC (B1; 559878), 1:50,
anti-CRTH2-AF647 (BM16; 558042), 1:25,
anti-CD16-BUV496 (3G8; 564653), 1:50,
anti-CD56-AF700 (B159; 557919), 1:100,
anti-CD7-PE-CF594 (M-T701; 562841), 1:50,
anti-CD7-BV711 (M-T701; 564018), 1:100,
anti-CD7-BV650 (M-T701; 740565), 1:100,
anti-CD25-BUV737 (2A3; 612806), 1:100,
anti-CD161-BUV395 (HP-3G10; 745737), 1:12.5,
anti-CD200R-BV421 (OX-108; 566344), 1:50,
anti-IFNg-BUV395 (B27; 624163), 1:200,
anti-IL-13-BV421 (JES10-5A2; 624124), 1:100,
anti-RORgt-AF647 (Q21-559; 563620), 1:50,
anti-T-bet-BV786 (O4046; 564141). 1:25,
Purchased from Thermofisher scientific:
anti-CD3-FITC (OKT3; 11-0037-41), 1:50,
anti-CD5-FITC (UCHT2; 11-0059-42), 1:50,
anti-TCRab-FITC (IP26; 11-9986-42), 1:50,
anti-CD127-PE-Cy7 (eBioRDR5; 25-1278-42), 1:100,
anti-IL-22-PE-Cy7 (22URTI; 25-7229-42), 1:25,
anti-EOMES-PE-Cy7 (WD1928; 25-4877-42/41), 1:25,
anti-EOMES-PE (WD1928; 12-4877-42), 1:50,
anti-GATA-3-PerCP-eFluor710 (TWAJ; 46-9966-42) 1:50.

Validation

All antibodies used were monoclonal, commercially available, and statements about the use and validation of all antibodies can be found on the manufacturers website (see Supp. Table 2). All antibodies were validated and quality controlled by the manufacturer for affinity to the specific target human protein by flow cytometry and possibly other applications. Specific details for individual antibodies can be found on the manufacturer's website. Examples of validation statements are provided below:

Thermo Fisher Scientific: This Antibody was verified by Relative expression to ensure that the antibody binds to the antigen stated.
Miltenyi Biotec : In order to compare the epitope specificity of an antibody, the clone being used is compared with other known clones recognizing the same antigen in a competition assay.
BioLegend: Antibody clones are then tested in a variety of assays to see which applications they are suited for.
BD Biosciences: The specificity is confirmed by using multiple applications that may include a combination of flow cytometry, immunofluorescence, immunohistochemistry or western blot to test a combination of primary cells, cell lines or transfectant models.

Eukaryotic cell lines

Policy information about cell lines

Cell line source(s)

OP9 ATCC CRL-2749, OP9-DLL4 Institut Pasteur

Authentication

None of the cell lines were authenticated

Mycoplasma contamination

Cells were visually assessed for the presence of extranuclear DNA via DAPI staining, none was observed.

Commonly misidentified lines
(See ICLAC register)

No commonly misidentified cell lines were used in this study

Human research participants

Policy information about studies involving human research participants

Population characteristics

Healthy donors were randomly selected (age and sex). The EFS (Établissement Français du Sang) did not share information about clinical history or past diagnosis. IL12RB1 and RORC patients were selected based on the diagnosis and genetic mutation but age and sex were selected for randomly. All patients had genetic etiology for MSMD and CMC as previously

	published, additional information about specific patients including age, sex and origin is provided in Supplementary Table 1. Healthy donors were provided without preselection and ranged in age from 19-61 with a 60-40 ratio of males to females.
Recruitment	Healthy donors were randomly recruited by EFS (Établissement Français du Sang). PBMC from patients was obtained from the Laboratory of Human Genetics of Infectious Diseases (Institut Imagine, Necker, Paris)
Ethics oversight	All studies required ethics approval from the institutional ethics committees. Patient recruitment was done under the ID-RCB 2010-A00650-39 and 2010-A00636-33 delivered by EC IDF-II, France.<br><br>Healthy donors were recruited by EFS and samples given to the Institut Pasteur under the agreement N°18/EFS/041.

Note that full information on the approval of the study protocol must also be provided in the manuscript.

Clinical data

Policy information about clinical studies

All manuscripts should comply with the ICMJE guidelines for publication of clinical research and a completed CONSORT checklist must be included with all submissions.

Clinical trial registration	*Provide the trial registration number from ClinicalTrials.gov or an equivalent agency.*
Study protocol	*Note where the full trial protocol can be accessed OR if not available, explain why.*
Data collection	*Describe the settings and locales of data collection, noting the time periods of recruitment and data collection.*
Outcomes	*Describe how you pre-defined primary and secondary outcome measures and how you assessed these measures.*

Dual use research of concern

Policy information about dual use research of concern

Hazards

Could the accidental, deliberate or reckless misuse of agents or technologies generated in the work, or the application of information presented in the manuscript, pose a threat to:

No	Yes	
☒	☐	Public health
☒	☐	National security
☒	☐	Crops and/or livestock
☒	☐	Ecosystems
☒	☐	Any other significant area

Experiments of concern

Does the work involve any of these experiments of concern:

No	Yes	
☒	☐	Demonstrate how to render a vaccine ineffective
☒	☐	Confer resistance to therapeutically useful antibiotics or antiviral agents
☒	☐	Enhance the virulence of a pathogen or render a nonpathogen virulent
☒	☐	Increase transmissibility of a pathogen
☒	☐	Alter the host range of a pathogen
☒	☐	Enable evasion of diagnostic/detection modalities
☒	☐	Enable the weaponization of a biological agent or toxin
☒	☐	Any other potentially harmful combination of experiments and agents

Flow Cytometry

Plots

Confirm that:

☒ The axis labels state the marker and fluorochrome used (e.g. CD4-FITC).

☒ The axis scales are clearly visible. Include numbers along axes only for bottom left plot of group (a 'group' is an analysis of identical markers).

☒ All plots are contour plots with outliers or pseudocolor plots.

☒ A numerical value for number of cells or percentage (with statistics) is provided.

Methodology

Sample preparation	Sample preparation is detailed in the materials and methods. Briefly, samples of human blood were isolated using a Ficoll-Paque gradient. For bulk sorting PBMCs were first depleted of T cells, B cells, and residual, monocytes, pDCs and erythrocytes using biotinylated antibodies (anti-CD3, anti-CD14, anti-CD19, anti-CD123, anti-CD235a) (Thermofisher Scientific), followed by incubation with MojoSort anti-biotin magnetic nanobeads (Stemcell) according to the manufacturer's instructions. Samples used for cell sorting and extracellular phenotyping were stained for 15 minutes at room temperature with antibodies against extracellular markers and viability dye efluor506 (Thermofisher Scientific) in 2% FCS - 1x PBS.<br><br>Samples used for intracellular staining of cytokines were stimulated for 3-4 hours with PMA, Ionomycin (Sigma) and Golgi plug (BD). Staining for transcription factors was performed using unstimulated cells. In brief, samples were stained for 30 minutes at 4 degrees for antibodies to extracellular surface markers and viability dye efluor506 (Thermofisher Scientific). Fixation and permeabilization was then performed using the FoxP3/Transcription Factor staining kit (BD) according to the manufacturer's instructions. Cells were then stained with intracellular and intranuclear markers for 30 minutes at room temperature.
Instrument	A BD LSR Fortessa was used for acquisition of data regarding functional (cytokine expression) and transcription factor expression. Cell sorting was performed on either a BD ARIA II, BD ARIA III or BD ARIA fusion, determined by availability on the platform, and the phenotype of the sample was concurrently acquired.
Software	All flow cytometery data was acquired using BD FACS DIVA v8, and analyzed using FlowJo (BD) v10
Cell population abundance	Cell populations in bulk were sorted to 99% purity. Single cell populations were sorted using index sorting and at single cell purity.
Gating strategy	The gating strategy for cell sorting and phenotyping is provided in Supplementary Figure 1. In brief, Lymphocytes were identified by FSC-A SSC-A, single cells were identified by the ratio of FSC-W vs FSC-H followed by SSC-W vs SSC-H. ILCPs were then identified by sequential gating of CD45+ live cells, Lineage-CD7+, CD94-CD127+, NKG2A-CD16- and finally CD117 +CRTH2-. NK cells were identified on the CD45+live, Lineage-CD7+ cells as CD94+CD16+. NK cell subsets were identified CD56 +CD127+ for CD56 Bright NK cells and CD56dimCD127- for CD56 dim NK cells.<br><br>The gating strategy for ICS analysis is provided in Supplementary figure one, examples of stainings are included in figures or supplementary figures. Clones and purified bulks were identified first by FSC-A SSC-A, single cells were identified by the ratio of FSC-W vs FSC-H followed by SSC-W vs SSC-H. Clones were then gated on CD45+live cells, and CD3- and identified as described in Supplementary Figure 4. Transcription factor and cytokine expression on bulks was further gated on EOMES-cells.

☒ Tick this box to confirm that a figure exemplifying the gating strategy is provided in the Supplementary Information.
